# Successful *in vitro* propagation of porcine bocavirus: Demonstrating dual respiratory-enteric tropism and pathogenicity

**DOI:** 10.1371/journal.ppat.1013631

**Published:** 2025-11-03

**Authors:** Zhaoyang Ji, Xin Zhang, Mei Xue, Jianfei Chen, Da Shi, Hongyan Shi, Hongliang Zhang, Liaoyuan Zhang, Tingshuai Feng, Xiaoyuan Zhu, Xiuwen Li, Dakai Liu, Mengting Wang, Miaomiao Zeng, Li Feng

**Affiliations:** State Key Laboratory for Animal Disease Control and Prevention, Harbin Veterinary Research Institute, Chinese Academy of Agricultural Sciences, Harbin, Heilongjiang, China; Pirbright Institute, UNITED KINGDOM OF GREAT BRITAIN AND NORTHERN IRELAND

## Abstract

The inability to propagate porcine bocavirus (PBoV) *in vitro* has severely impeded research into its fundamental biology and pathogenic potential since its discovery 15 years ago. This study reports the successful isolation and characterization of a novel PBoV strain, PBoV-CNH, from diarrheic piglets in China. Crucially, PBoV-CNH was isolated and propagated in LLC-PK1 cells, a kidney-derived cell line from 3-4-week-old pigs, matching the age of the susceptible host. This represents the first documented isolation of PBoV in a continuous cell line. The isolate exhibited typical bocavirus morphology (20–30 nm particles), shared 94.15% whole-genome nucleotide identity with the NCBI reference strain (NC_016031.1), and displayed hemagglutination activity (HA) characteristic of *Parvoviridae*. Phylogenetic analysis revealed that PBoV-CNH clusters within a clade containing human bocaviruses (HBoVs), highlighting close genetic relatedness. Experimental infection of piglets confirmed PBoV-CNH as a primary pathogen. Notably, the virus exhibited dual tissue tropism: orally infected piglets developed acute diarrhea with high intestinal viral loads, while intranasally infected piglets showed diarrhea, significant lung pathology, and the highest viral loads in respiratory tissues. This efficient replication and shedding in the respiratory tract, combined with phylogenetic proximity to HBoVs and a previously reported human-PBoV infection case, signals a tangible risk of cross-species transmission. To our knowledge, this work constitutes the first successful *in vitro* propagation of PBoV and provides definitive experimental evidence, fulfilling Koch’s postulates, of its *in vivo* pathogenicity and tissue tropism. These findings provide essential tools and foundational insights for future research into PBoV biology, transmission, and control strategies.

## Author summary

This study overcomes the 15-year barrier to PBoV research by achieving its first successful isolation and propagation in LLC-PK1 cells, providing an essential *in vitro* tool. Critically, experimental infection of naïve piglets establishes PBoV as a primary pathogen capable of causing both gastrointestinal and respiratory disease, with tropism dictated by infection route. The finding of high viral loads and pathology in respiratory tissues, combined with phylogenetic clustering of PBoV-CNH alongside HBoVs, signals a tangible risk for cross-species transmission. This work provides the fundamental platform and insights urgently needed to investigate PBoV biology, transmission dynamics, and zoonotic potential, enabling future mitigation strategies.

## Introduction

Bocaviruses have been detected in diverse species including humans [1–4], cattle [5,6], canines [7,8], gorillas [9,10], felines [11], sea lions [12], and potentially other hosts. PBoV, a new member of the *Bocaparvovirus* genus, was discovered in Sweden in 2009. [13]. PBoV exhibits global distribution and has been identified in pigs presenting with both diarrheal and respiratory manifestations [14,15]. Despite its worldwide prevalence, understanding of PBoV’s pathogenic mechanisms and biological characteristics remains limited. This knowledge gap primarily stems from persistent challenges in establishing reproducible *in vitro* cultivation systems despite substantial research efforts [16–18].

As a single-stranded DNA virus within the *Bocaparvovirus* genus, PBoV demonstrates associations with respiratory and enteric disease. Its genome contains three open reading frames (ORFs): ORF1 encodes the nonstructural protein NS1; ORF2 encodes viral capsid proteins VP1 and VP2 (with VP1 containing the complete VP2 sequence plus an N-terminal extension); and ORF3 encodes nuclear phosphoprotein NP1, which suppresses type I interferon production [19–21].

Based on the sequence analysis of VP1, PBoVs are phylogenetically classified into three major genotypes [22]. Genotypes 2 (G2) and G3 form distinct monophyletic clades comprising strains from China, Kenya, and Europe, while G1 includes isolates from Asia, Europe, and the USA. This distribution underscores the global genetic diversity of PBoV and its tendency toward geographical clustering [23].

Epidemiological studies reveal significant PBoV prevalence: Analysis of 203 tissue samples from pigs with respiratory or diarrheal symptoms across North America showed a 43.3% positivity rate, increasing to 48.8% in diarrheic specimens [24]. Similarly, examination of 340 porcine fecal samples from five Chinese provinces demonstrated regional prevalence rates of 45–75% [25]. High rates of co-infection with other swine pathogens—including porcine circovirus type 2 (PCV-2), porcine reproductive and respiratory syndrome virus (PRRSV), classical swine fever virus (CSFV), and other enteric viruses—are frequently observed [15,24]. Notably, PBoV detection in a child with acute respiratory infection [26] and in murine rodents/house shrews (via throat swabs, feces, and serum) [27] suggests potential zoonotic transmission. Nevertheless, bocavirus pathogenicity across species remains ambiguous, with unresolved questions regarding its role as primary pathogens, opportunistic agents, or innocent bystanders.

The inability to culture PBoV *in vitro* and its frequent co-detection with other swine viruses have impeded characterization of its biological features and pathogenic role. Propagation attempts in various cell lines—including porcine alveolar macrophages (PAM), Human embryonic kidney 293 T (HEK293T), MARC-145, swine testicular (ST), and porcine kidney epithelial (PK-15)—have proven unsuccessful [16]. While two strains were isolated from primary porcine kidney cells of postweaning multisystemic wasting syndrome (PMWS)-affected pigs in Northern Ireland [28], representing the sole reported *in vitro* cultivation success, no *in vivo* pathogenicity assessment followed. The absence of robust culture systems and animal models has hindered definitive classification of bocaviruses as respiratory or enteric pathogens in humans, swine, and other species.

This study reports the successful isolation of strain PBoV-CNH in continuous cell lines and characterization of its replication kinetics and pathogenicity. Phylogenetic analysis places CNH within the highly diverse recently designated Group 3 clade, demonstrating robust replication in LLC-PK1 cells. Challenge studies confirmed PBoV-CNH’s high pathogenicity, inducing acute diarrhea and pulmonary pathology in pigs. These findings parallel unresolved questions regarding human bocavirus (HBoV) pathogenicity, where research is similarly constrained by inadequate culture systems and animal models [29]. Our work systematically defines PBoV’s biological properties and pathogenicity in swine, advancing molecular insights into its infection dynamics and tissue tropism.

## Results

### Global distribution and milestone events in PBoV research

**Fig 1:**
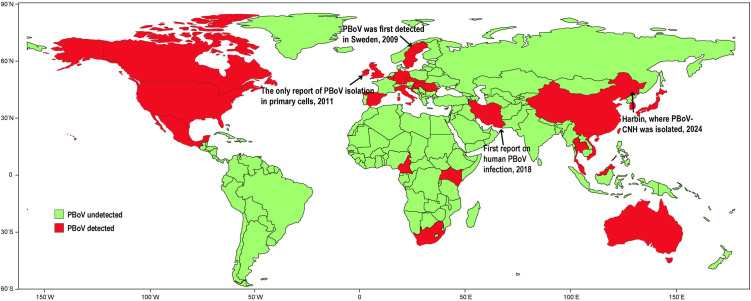
Global distribution of PBoV-positive samples and four major events following its initial detection. The base map was created using public domain data from Natural Earth (https://www.naturalearthdata.com/downloads/110m-cultural-vectors/).

Since its initial detection in Sweden in 2009, PBoV has demonstrated a global distribution, with positive samples identified across multiple countries. Four key events have marked PBoV research (Fig 1): (a) Initial detection of PBoV in Sweden, 2009; (b) First isolation of PBoV in primary cell cultures two years post discovery—the only successful isolation reported to date; (c) A report on PBoV infection in a three-year-old child presenting acute respiratory symptoms, highlighting potential zoonotic risks; and (d) the 2024 identification of PBoV in diarrheic weaned piglets of unknown etiology, notably accompanied by the successful isolation in continuous cell lines in present study, advancing tools for its virological characterization.

### Next-generation sequencing analysis identified PBoV in a diarrhea outbreak

**Fig 2:**
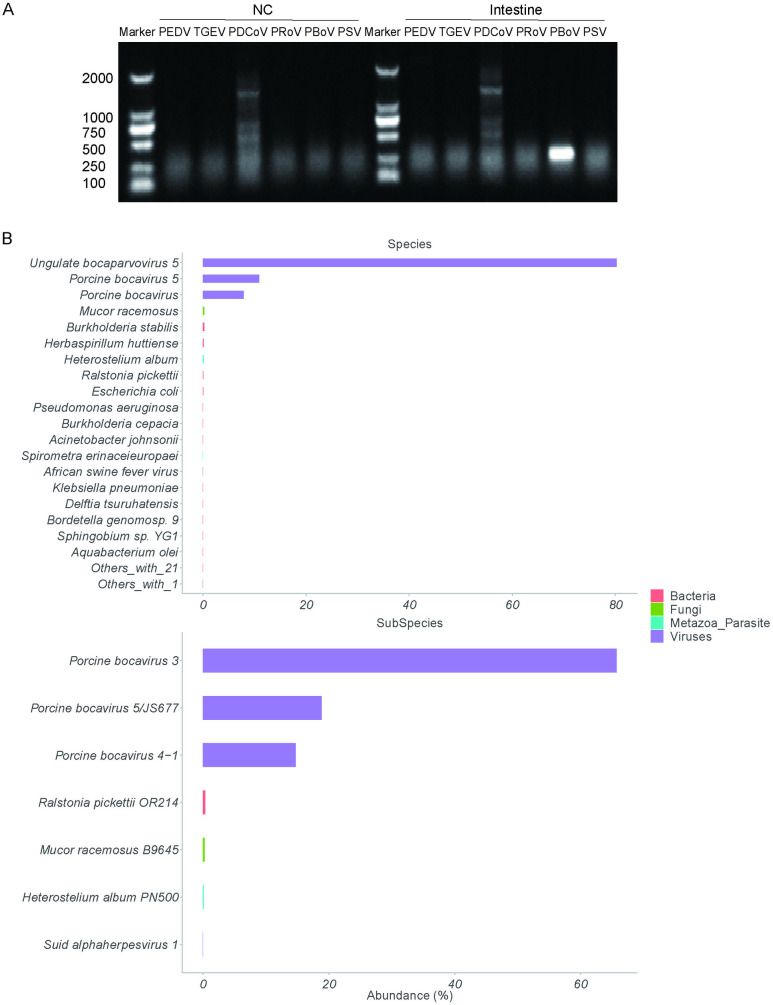
NGS analysis identified PBoV as the likely causative agent in an outbreak of diarrhea of unknown origin. (A) PCR results confirmed that PBoV existed in intestine samples. (B) NGS statistical results revealed bocavirus as the only identified virus in diarrhea samples.

In April 2024, a diarrhea outbreak with unidentified causative agents occurred on a pig farm in Harbin, China, weaned piglets aged 30 days suffered from watery diarrhea and dehydration. Notably, PCR analysis of intestine samples ruled out common swine enteric pathogens, including PEDV, TGEV, PDCoV, PoRV, and PSV (Fig 2A), but was positive for PBoV. To investigate the potential involvement of undetected pathogens, fecal samples were subjected to **next-generation sequencing** (NGS) analysis. This comprehensive approach, coupled with a search against the NCBI NT database, aimed to uncover any potentially associated microbial entities. The NGS analysis yielded a total of 878,500 clean reads. Among these, 481,076 reads were classified as viral sequences. Of these viral reads, 481,018 were specific sequences of *Bocaparvovirus*, close to 100% (Fig 2B). This analysis further confirmed that PBoV might be the main causative pathogen responsible for the diarrhea outbreak.

Collectively, these findings indicate a potential association between PBoV and the diarrhea epidemic and highlight the need for further investigation into the pathogenic role of PBoV.

### Cell isolation and *in vitro* characterization of PBoV

**Fig 3:**
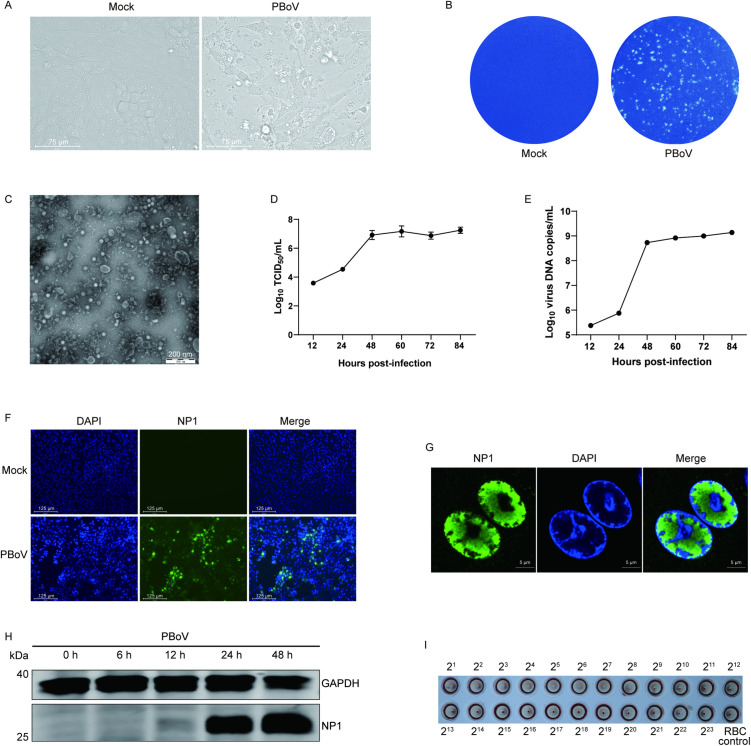
Cell isolation and *in vitro* characterization of PBoV. (A and B) Virus-induced CPE in PBoV-infected LLC-PK1 cells that are visible on light microscopy (A) and in plaque assays (B). Scale bar represents 75 μm. (C) Negatively stained virions purified from PBoV-infected LLC-PK1 cells. Scale bar represents 200 nm. (D and E) Growth curves of PBoV in LLC-PK1 cells, with titers peaking mostly at 48 to 60 hpi determined by TCID_50_, viral DNA copies peaking at 84 hpi determined by qPCR. (F) PBoV-infected LLC-PK1 cells detected by IFA, the infected cells were stained with a mouse serum raised against the PBoV NP1 protein. Scale bar represents 125 μm. (G) Nuclear localization signal of NP1 protein was observed in PBoV-infected cells. Scale bar represents 5 μm. (H) The expression levels of NP1 protein increased in a time-dependent manner in PBoV-infected cells. (I) PBoV propagated in LLC-PK1 cells can agglutinate 0.6% fresh guinea pig red blood cells with a HA titer of 2^8^.

Previous attempts to culture bocavirus *in vitro* using various cell lines (including HEK293T, MARC145, ST, and PK15) failed to support virus propagation [16]. Here, given that PBoV mainly infects piglets within 4 weeks of age in pig farms, we utilized the LLC-PK1 cell line for virus isolation, this cell line was derived from the kidney of a 3–4-week-old male pig, distinguishing it from PK15 cells, which are derived from the kidney of an adult pig. LLC-PK1 cells were inoculated with clarified supernatant from homogenized PBoV-positive intestinal samples. Cytopathic effect (CPE) was observed by passage 2 (day 4 post-inoculation) and became consistent through subsequent passages (Fig 3A) with the formation of obvious plaques (Fig 3B). Transmission electron microscopy revealed numerous 20–30 nm round particles (Fig 3C), exhibiting morphological characteristics typical of viruses within the *Parvovirinae* subfamily. Viral growth kinetics demonstrated an initial exponential increase, reaching a plateau at 60 hours post-infection (hpi) (Fig 3D). Meanwhile, viral DNA levels began rising at 12 hpi and continued increasing steadily until 84 hpi (Fig 3E).

PBoV ORF3 encodes NP1 protein, a unique non-structural protein specific to bocaviruses, which is absent in other members of the *Parvovirinae* subfamily. To confirm successful isolation, NP1 protein was expressed and purified, and a mouse-derived polyclonal antibody (pAb) against NP1 was generated (S1 Fig). Indirect immunofluorescence assay (IFA) using this anti-NP1 pAb confirmed PBoV replication in LLC-PK1 cells (Fig 3F). Furthermore, a non-classical nuclear localization signal (ncNLS) was observed in infected cells (Fig 3G), resembling the characteristic of NP1 in HBoV [20]. Western blotting confirmed a time-dependent increase in NP1 protein expression levels (Fig 3H). PBoV propagated in LLC-PK1 cells exhibited HA, agglutinating 0.6% fresh guinea pig red blood cells with a titer of 2^8^ (Fig 3I). This characteristic is consistent with PPV, another member of the *Parvoviridae* family known for HA.

Collectively, these results confirm the isolation of replicating PBoV in LLC-PK1 cells. This isolate is designated PBoV/CNH/2024 (abbreviated to CNH; GenBank accession no. PQ631047).

### Genomic characteristics and phylogeny of PBoV-CNH

**Fig 4:**
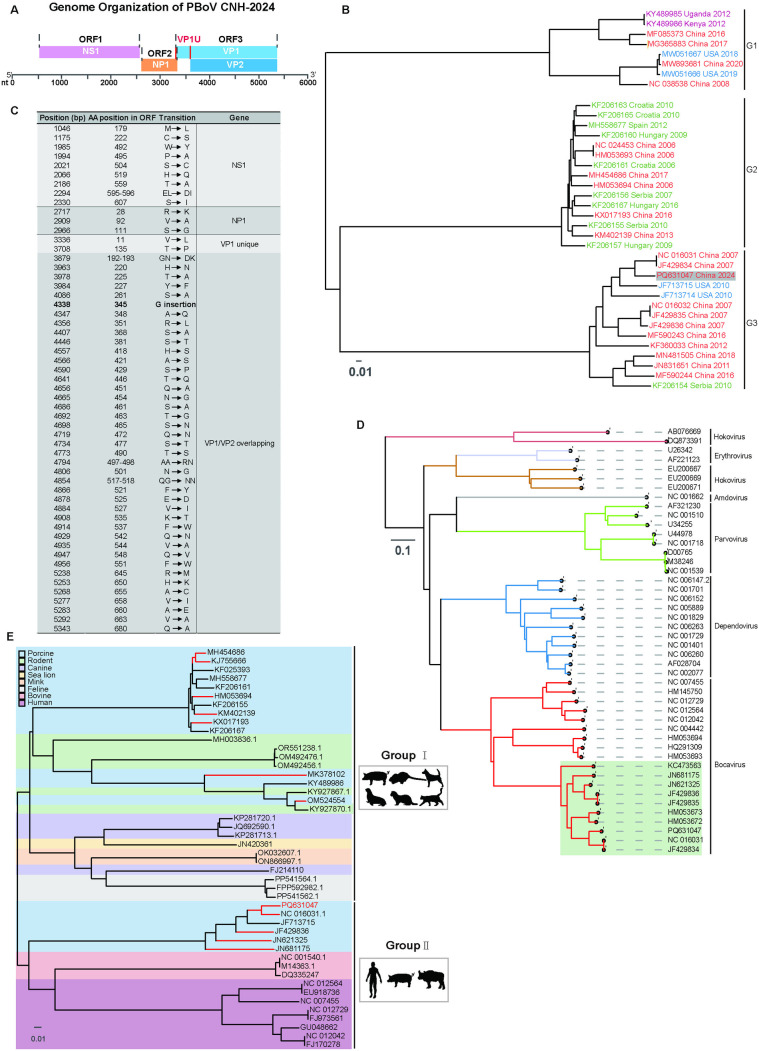
Genomic characteristics and phylogeny of PBoV-CNH. (A) The genome organization of PBoV-CNH. The diagram is drawn to scale, and the scale bar is shown at the bottom. The abbreviation nt denotes nucleotides. (B) Phylogenetic analysis of PBoV isolates based on the VP1 gene. This analysis employed a neighbour-joining method using the MEGA 6.0 software. Bootstrap probabilities for each node were calculated using 1,000 replicates. The gray background highlights the PBoV isolated in this study. Scale bars indicate the number of nt substitutions per site. The virus strains detected in China, Europe, America, Africa are highlighted in red, green, blue and purple, respectively. (C) Summary table of amino acid coding differences between PBoV and NCBI reference strain NC_016031. (D) Phylogenetic trees of the VP1 gene sequence of PBoV-CNH in the present study and corresponding sequences of other parvoviruses. The green background highlights the PBoV strains. Scale bars indicate the number of nt substitutions per site. The tree was constructed by MEGA software, with neighbour-joining method, 1,000 bootstrap replications. (E) Phylogenetic analysis of complete genome sequences of bocavirus from different species. Species are colored by Porcine (blue), Rodent (green), Canine (light purple), Sea lion (yellow), Mink (orange), Feline (gray), Bovine (pink), Human (purple), PBoV strains isolated in China are highlighted in red branches. Scale bars, nt substitutions per site.

The PBoV-CNH genome is 5,484 nt long and exhibits the typical bocavirus organization, containing three open reading frames (ORFs) (Fig 4A): ORF1 encodes the non-structural protein NS1; ORF2 encodes the structural proteins VP1 (containing a unique 408-bp N-terminal region, VP1u) and VP2; and ORF3 encodes NP1. Phylogenetic analysis based on the VP1 nt sequence placed PBoV-CNH within G3, which predominantly comprises isolates from China (Fig 4B). BLAST analysis of the complete genome revealed high sequence identity with other PBoVs, sharing the highest nt identity (~94.15%) with the NCBI reference strain NC_016031.1 (detected in Hong Kong, 2011). Comparative amino acid analysis identified multiple substitution mutations and one insertion mutation relative to this reference strain, with the highest sequence divergence observed within the VP1 gene (Fig 4C).

To determine the phylogenetic position of PBoV within the *Parvovirinae* subfamily, an analysis was performed using near full-length VP1 sequences (n = 45) representing different genera (*Parvovirus, Amdovirus, Bocavirus, Erythrovirus, Dependovirus, Hokovirus*). The resulting tree showed distinct branches corresponding to each genus (Fig 4D). PBoV-CNH clustered within the *Bocavirus* genus branch alongside CnMV (NC_004442), GBoV (HM145750), and HBoV strains (NC_012729.2, NC_012564.1, NC_012042), confirming its close evolutionary relationship to other bocaviruses and distinct separation from other genera. To further clarify relationships within the *Bocaparvovirus* genus, a phylogenetic tree was constructed using complete genome sequences of PBoV strains and recognized *Bocaparvovirus* species (human, porcine, rodent, canine, sea lion, mink, feline, bovine). This analysis revealed segregation into two major clades (Fig 4E). PBoV strains were distributed across both clades, with PBoV-CNH clustering specifically within the clade containing HBoVs and BBoVs. PBoV-CNH is highlighted in red, and red branches indicate PBoVs isolated in China.

These findings confirm that the CNH strain possesses characteristic features of bocaviruses. The observed clustering warrants vigilance regarding the potential zoonotic risk associated with PBoV.

### Pathogenicity of PBoV-CNH in piglets

Due to the lack of cell-cultured bocavirus, the pathogenicity of PBoV has not been determined to date, and it is still unclear whether bocavirus are respiratory or enteric pathogens. To characterize the pathogenicity of PBoV-CNH, fifteen 3-day-old neonatal piglets (negative for PEDV, TGEV, PDCoV, SADS-CoV and PBoV) were used. Five piglets were orally inoculated with PBoV-CNH at a dose of 1 × 10^5^ TCID_50_, five were intranasally inoculated with the same dose, and five control piglets were inoculated orally and intranasally with DMEM.

**Fig 5:**
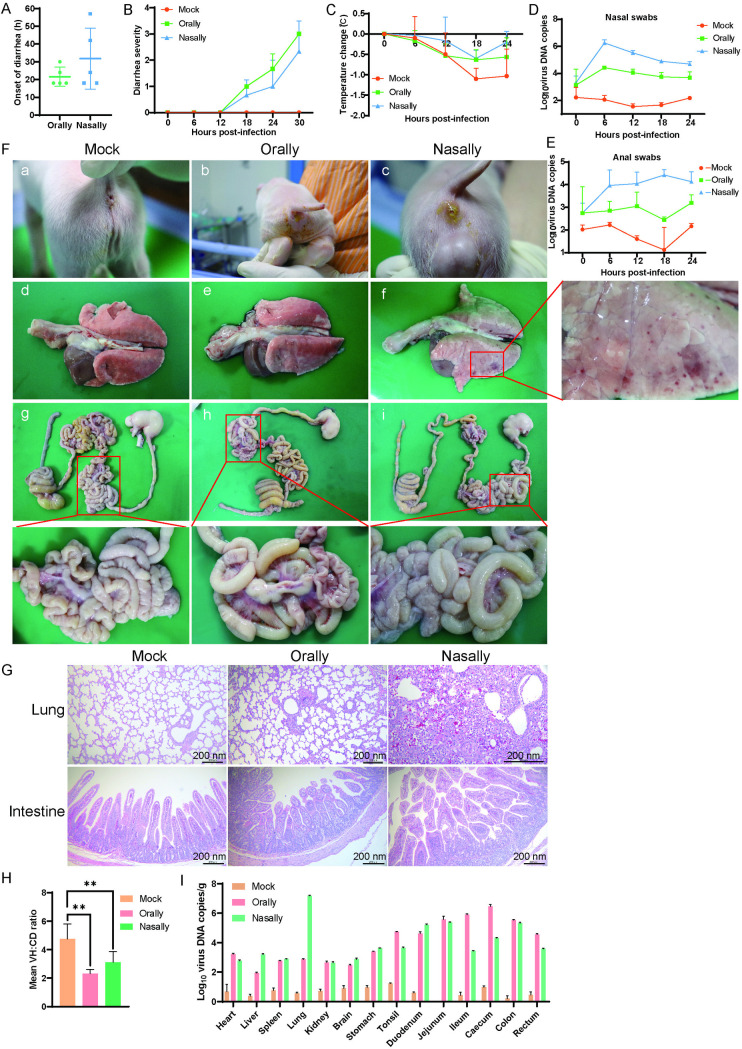
Pathogenicity of PBoV-CNH in piglets. (A-C) Onset of diarrhea (A), diarrhea severity scores (B) and body temperature change (C) were documented once every six hours. (D and E) Viral DNA copies in fecal and nasal swabs were collected for the detection of virus shedding time by qPCR. (F) (a, b and c) 3-day old piglets were orally- and intranasally-inoculated with PBoV-CNH (1 × 10^5^TCID_50_/pig) exhibited diarrhea symptom, watery diarrhea was observed in both orally and intranasally-infected piglets; (d, e and f) there are bleeding spots in the lungs of intranasally-infected piglets; (g, h and I) pathological changes were observed in intestines of challenged piglets. (G) Histopathological changes in the intestines and lungs of piglets challenged with PBoV. (H) Morphometric analysis for VH:CD ratio for the villous atrophy. P-value < 0.01 was considered as significant (**P < 0.01). (I) Piglets were euthanized, and different organs were collected for viral load determinations.

Clinical signs, including body temperature, diarrhea, and respiratory distress, were monitored. Anal and nasal swabs were collected every 6 h to assess virus shedding. Diarrhea severity was quantified using a 5-point scale (0 = healthy, 1 = soft but formed feces, 2 = mild diarrhea, 3 = watery diarrhea, 4 = watery diarrhea plus vomiting). Diarrhea onset occurred within 18–30 hpi in orally infected pigs and 18–56 hpi in intranasally infected piglets (Fig 5A). Both infected groups exhibited mild to watery diarrhea by 30 hpi, with scores documented in Fig 5B. In the intranasally infected group, rectal temperature increased by 0.67°C starting at 18 hpi, peaking at +1.0°C by 24 hpi. Orally infected piglets showed a 0.47°C rise from 18 hpi, peaking at +0.53°C by 24 hpi (Fig 5C). High levels of PBoV shedding were detected in both nasal and anal swabs from infected piglets (Fig 5D and 5E), indicating efficient virus shedding into the environment and potential for transmission via respiratory and digestive routes.

Piglets were euthanized for pathological examination upon development of clinical signs in all infected groups. Challenged piglets exhibited typical pathological changes, including intestinal wall thinning, luminal gas distension, and mesenteric hemorrhage. Three out of five intranasally infected piglets showed elevated nasal secretions, and two out of five exhibited pulmonary petechiae (Fig 5F), suggesting potential respiratory tract involvement and multiple shedding routes. Histopathological examination revealed pulmonary inflammation in intranasally infected piglets. Both orally and intranasally infected piglets displayed marked villus atrophy in the intestine, (Fig 5G), consistent with the morphometric analysis for villous height/crypt depth (VH:CD) ratio (Fig 5H). Quantitative PCR (qPCR) detected viral DNA in tissues from challenged piglets. Notably, virus was primarily localized to the intestines in orally inoculated piglets, but was found in both intestines and lungs of intranasally inoculated piglets (Fig 5I). The highest viral DNA copy numbers were detected in the lungs of intranasally infected piglets and the intestines of orally infected piglets, with minimal detection in stomach, tonsil, spleen, kidney, liver, inguinal lymph nodes, or heart.

Furthermore, PBoV distribution within the gastrointestinal tract differed from common swine enteric coronaviruses (PEDV, PDCoV and TGEV), as it was detected throughout the intestines rather than being restricted to the jejunum and ileum. This suggests a potentially distinct pathogenic mechanism. These results demonstrate that PBoV is pathogenic to pigs, capable of causing both gastrointestinal and respiratory tract infections.

### Susceptibility of various cell lines to PBoV-CNH

Trypsin is known to facilitate the invasion of several swine enteric coronaviruses (PEDV, PDCoV and SADS-CoV) [30–32] and is often critical for successful virus isolation. Therefore, we investigated the effect of trypsin on PBoV replication in LLC-PK1 cells.

**Fig 6:**
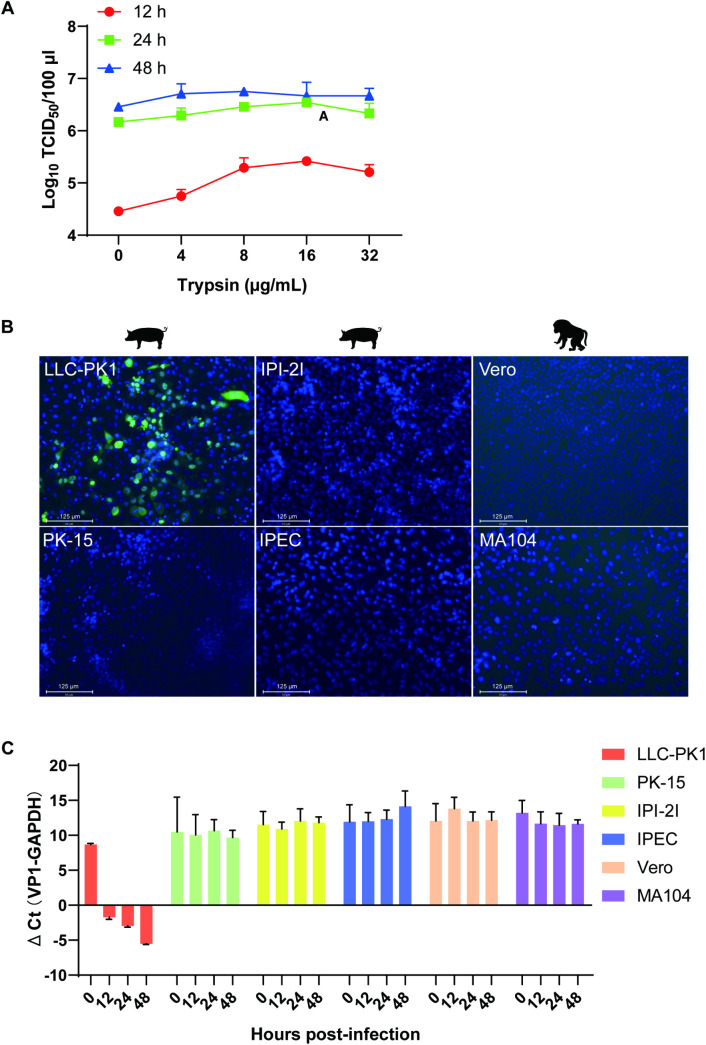
Susceptibility of various cell lines to PBoV-CNH. (A) Effect of trypsin on PBoV replication in LLC-PK1 cells. Virus titers were detected following trypsin treatment at doses of 0, 4, 8, 16, and 32 μg/mL at 12, 24, and 48 hpi. (B) PBoV-infected different cells were detected by IFA. The cells were infected with PBoV-CNH (MOI = 1) in the presence of 16 μg/mL trypsin, and then incubated with mouse polyclonal antibodies against the PBoV-NP1 protein. Scale bar represents 125 μm. (C) Growth kinetics of PBoV in different cell lines. Supernatants were collected at the indicated time points (0, 12, 24 and 48 hpi), and cycle threshold (CT) values for viral DNA copies were measured at each time point using qPCR. Mean ± SD (error bars) are shown (n = 3).

A dose-dependent increase in virus titer was observed in the presence of trypsin. The peak enhancement (a 10-fold increase) occurred at 16 μg/mL trypsin at 12 hpi, with no significant effect observed at 24 or 48 hpi (Fig 6A). These results suggest that trypsin facilitates PBoV infection during the early stages of replication and may play a pivotal role in successful virus isolation *in vitro*.

The longstanding inability to culture PBoV in vitro has significantly hindered research into its biological characteristics. To determine whether the successful isolation of the PBoV-CNH strain was due to the use of the LLC-PK1 cells, we compared its replication in other commonly used porcine and monkey cell lines, including PK-15, porcine ileal epithelial (IPI-2I), intestinal porcine epithelial (IPEC) African green monkey kidney (Vero) and rhesus monkey kidney (MA104) cells in the presence of 16 μg/mL trypsin, the concentration found to be optimal for PBoV replication as described above. However, no viral replication was detected in any of these cell lines by IFA (Fig 6B). Furthermore, qPCR targeting the VP1 gene confirmed the absence of efficient viral replication (Fig 6C). These results suggest that the successful isolation of PBoV-CNH may be due to the specific permissiveness of the LLC-PK1 cell line.

## Discussion

PBoV was first identified in 2009 in Swedish pigs affected by PMWS [13]. Although PBoV has been detected globally in subsequent years, the inability to isolate the virus in cell culture has significantly hampered understanding of its fundamental biology, including pathogenicity, infectivity, and transmission mechanisms. This study addresses this critical gap by reporting the successful isolation of PBoV from rectal swabs of diarrheic pigs, using the LLC-PK1 cell line derived from the kidney of a 3–4-week-old pig–an age matches the susceptible host population. The pronounced CPE, high virus titers, and robust immunofluorescence signal observed in infected LLC-PK1 cells demonstrate the high permissiveness of this cell line to PBoV infection. This breakthrough isolation provides an essential tool for future studies on PBoV pathogenesis, receptor identification, gene function, and antiviral strategies.

Previous attempts to propagate PBoV in various cell lines, including HEK293T, MARC-145, PK15, PAM, and ST cells, were unsuccessful [16]. The choice of LLC-PK1 cells, originating from tissue developmentally matched to the age of susceptible host, proved pivotal. Our animal pathogenicity studies, enabled by this isolate (PBoV-CNH), demonstrated that PBoV replicates efficiently and causes disease in pigs. Piglets infected via oral or intranasal routes developed clinical signs including diarrhea and pyrexia. Critically, orally infected piglets exhibited intestinal tropism and diarrhea, while intranasally infected piglets showed significant viral replication and pathology in the lungs, alongside diarrhea. This dual tropism–infection and pathology in both the gastrointestinal and respiratory tracts–is a key finding. The detection of high viral loads in nasal swabs and lungs of intranasally infected piglets, coupled with respiratory pathology, suggests potential for respiratory shedding and warrants investigation into airborne transmission risk. The occurrence of diarrhea in intranasally infected piglets may result from the virus accessing the digestive tract.

Our phylogenetic analysis placed the isolate PBoV-CNH within G3 (predominantly Chinese strains) and revealed clustering within a clade containing HBoVs. While PBoV detection has been reported in a child with respiratory infection [26], this phylogenetic proximity underscores the need for further research to evaluate the potential for interspecies transmission. Analysis of the VP1 capsid protein, a known hotspot for variation, revealed mutation patterns consistent with natural evolutionary processes observed within the *Bocaparvovirus* genus, without evidence of strong immune-driven selection in the sequences examined.

A notable distinction between PBoV and common swine enteric coronaviruses is its distribution throughout the entire intestinal tract, as opposed to restriction to the jejunum and ileum. This widespread intestinal detection, observed alongside villus atrophy, suggests a potentially unique pathogenic mechanism compared to established enteric coronaviruses. While other studies have reported PBoV detection in diverse tissues like lymph nodes, tonsils, and spleen, indicating potential systemic spread [33], our qPCR analysis in experimentally infected neonates primarily localized the virus to the intestines and lungs, viral nucleic acids were detected only at minimal levels in other tissues. Tissue tropism may correlate with viral genotype, infection route, or the acute stage examined in our study.

The historical difficulty in isolating PBoV, often compounded by frequent co-infections with pathogens like PCV2, PEDV, and PRRSV [15,24], has obscured its primary pathogenic role. Clinically, PBoV shows a high co-infection rate with known immunosuppressive viruses such as PCV2 and PRRSV [34–36]. Given that PBoV itself also encodes an immunosuppressive mechanism—specifically, its NP1 protein significantly inhibits type I interferon (IFN-α/β) production via the TLR signaling pathway [37,38], we speculate that PBoV may exploit this immunosuppressive state caused by such viruses to achieve more efficient self-replication. While the high prevalence of PEDV in diarrheic pig populations also contributes to its frequent detection with PBoV, the specific interaction between PEDV and PBoV requires further investigation.

Our successful isolation and subsequent experimental infection studies provide compelling evidence establishing PBoV-CNH as a primary pathogen capable of causing clinical disease in pigs independently. The demonstration of its dual tropism clarifies previous ambiguities regarding its classification as solely an enteric or respiratory pathogen.

In conclusion, this study reports the groundbreaking isolation of PBoV in cell culture using the permissive LLC-PK1 line. We provide definitive experimental evidence establishing PBoV as a primary pathogen in pigs, capable of causing both gastrointestinal and respiratory tract infections, with tropism influenced by the route of inoculation. This work resolves longstanding questions about PBoV’s pathogenic potential and provides the essential foundation and tools (the isolate and a permissive cell line) for future mechanistic studies on viral entry, replication, pathogenesis, and transmission, ultimately informing the development of effective prevention and control strategies.

## Materials and methods

### Ethics statement

The procedures for sampling and processing pigs or mouse were reviewed and approved by the Animal Ethics Committee of the School of Harbin Veterinary Research Institute of the Chinese Academy of Agricultural Sciences. The Animal Ethics Committee approval number was 240904–01-GR and 240705–01-GR. All animals were performed in accordance with animal ethics guidelines and approved protocols.

### Sample collection and virus isolation

In April 2024, fecal swab samples were collected from 4-week-old piglets displayed diarrhea, and stored at the Harbin Veterinary Research Institute. As expected, a number of common porcine diarrhea viruses including PEDV, TGEV, PDCoV, SADS-CoV and PoRV were negative by PCR.

Samples were mixed with phosphate-buffered saline (PBS; pH 7.2). The suspension was then vortexed and centrifuged at 5, 000 × g for 5 min at 4 °C, supernatant was harvested and filtered using a 0.22-μm filter (Millipore, Billerica, MA), diluted 1:10 with serum-free DMEM, and a confluent monolayer of LLC-PK1 cells in 6-well plates was prepared for virus isolation. Before inoculation with intestine samples, cells were washed twice with PBS and 2 mL of the suspension supplemented with 8 μg/mL trypsin (Gibco) was seeded into LLC-PK1 cells. Following incubation at 37 °C for 45 min, the inoculum was removed and replaced with DMEM containing 8 μg/mL trypsin. The inoculated cells were maintained at 37 °C under 5% CO_2_ and observed daily for CPE for 5 days. For blind passage, cultures were harvested and used to inoculate fresh cells at a volume ratio of 1:5. This process was repeated for several passages until consistent CPE was observed. Notably, if CPE appears within 24 h, it is likely due to excessive inoculation dose or cell damage caused by toxic metabolites in the stool. In such cases, the inoculation dose can be reduced. Finally, the viral cultures were then subjected to NGS.

### Next-generation sequencing analysis

Samples that showed CPE after propagation in cells were subjected to next-generation sequencing (NGS). NGS analysis were performed to detect viral nucleotide (nt). The sequencing library was constructed using Ion Total RNA-Seq Kit v2 (Thermo Fisher Scientific). Libraries underwent sequencing on the Ion S5 sequencer provided by Thermo Fisher Scientific. Subsequently, a comprehensive analysis pipeline was employed to process the sequencing data, incorporating distinct steps to ensure rigorous evaluation: (a) Quality Filtering of Raw Data; (b) Host Genomic Sequence Removal; (c) BLASTn Search Against Viral Nucleotide Database; (d) BLASTx Search Against Viral Protein Database with DIAMOND v.0.9.0; (e) Contig Assembly and Additional BLASTx Search.

To facilitate viral complete genome sequencing, amplicon primers designed utilizing the Thermo Fisher Scientific online platform, with the PBoV (GenBank accession: NC_016031) genomes serving as references. Subsequently, we crafted sequencing libraries employing the NEBNext Ultra II DNA Library Prep Kit for Illumina, and these libraries were then sequenced on a MiSeq sequencer. To fill gaps in the genome sequences, PCR and Sanger dideoxy sequencing were conducted. CLC Genomic Workbench v9.0 was employed to assemble genome sequences. Additionally, 5′-RACE was utilized to determine accurate sequence of 5′-end with the SMARTer RACE 5′/3′ Kit (Takara).

### Growth curve

For analysis of the kinetics of PBoV replication in LLC-PK1 cell line, confluent cell monolayers in 12-well plates were infected with PBoV (MOI = 1) per well with three replicates for each cell line. Inoculation in 37°C for 2 h, then washed cells for three times with PBS. After incubated for indicated time points, frozen the plates and then centrifuged at 1,000 × g for 3 min to harvest the supernatant for detecting virus titers and DNA copies.

### Phylogenetic analysis

PBoV genome sequences and other reference sequences obtained from GenBank were aligned using MAFFT version 7 in BioAider V1.527 with default parameters and manually adjusted in Molecular Evolutionary Genetics Analysis (MEGA 6.0) software. Phylogenetic analysis based on the whole genome and VP1 gene sequence were constructed using a neighbour‒joining method with the phylogeny analysis function of MEGA software with the bootstrap value of 1,000 replicates.

### Preparation of polyclonal antibody against NP1 protein

The PBoV NP1 gene was amplified and inserted into pCold Ⅰ vector (GenBank number AB186388) for prokaryotic expression. Transformed Escherichia coli were grown at 37 °C until OD_600_ reaches 0.4-0.5, the culture was cooled to 15°C on ice for 30 minutes, followed by shaking at 15°C for 24 h in the presence of 1 mM IPTG. Bacteria were collected by centrifugation and suspended in 30 mL lysis buffer and lysed by sonication. The lysate, from which NP1 protein expression was confirmed with an anti-His-tag antibody, was applied to Ni2 + resin (Thermo Fisher Scientific). The purified NP1 protein was used to immunize mouse for preparation of antibody. After immunization and two boosts, mouse were euthanized to collect serum pAb. Mouse anti-NP1 protein serum was used to western blotting and IFA identification of PBoV.

### Animal infection studies

The animal infection experiments were conducted in strict accordance with the guidelines outlined in the National Institutes of Health’s Guide for the Care and Use of Laboratory Animals. The ethical utilization of animals in this research was granted official approval by the Committee of Animal Experiments at Harbin Veterinary Research Institute, under the approval number 250124–01-GR.

Fifteen 3-day-old specific pathogen free piglets were randomly divided into three groups, and piglets were tested to be free of PEDV, PDCoV, PoRV, CSFV, PCV2 and PPV infections using PCR. Piglets were orally-or intranasally-challenged with 1 × 10^5.0^ TCID_50_ of PBoV-CNH, while control group were orally- and intranasally-challenged with DMEM. Every group of piglets were raised in a biosafety level 2 laboratory, which could provide a comfortable environment without exogenous viruses. Clinical signs of the piglets in three groups were recorded every 6 h, including vomit, diarrhea and body temperature. Fecal and nasal swabs were collected every 6 h to determine the onset time of virus shedding. At the experiment endpoint, piglets were humanely euthanized and examined for pathology. Pictures were taken to record gross pathological changes to the intestines, lungs and other organs. To determine the viral loads in different tissues after treatment with the PBoV-CNH, 15 tissues were used to detect viral DNA copies by qPCR.

### Histopathology examination

The intestinal tissues and lungs were collected and immediately fixed with 4% paraformaldehyde for 24 h. The fixed intestinal samples were dehydrated, cleared in xylene, embedded in paraffin wax, sectioned, fixed on a glass slide, and stained with hematoxylin and eosin for histopathological examination.

## Supporting information

S1 Fig Preparation of a mouse-derived pAb to PBoV NP1 protein. (A) Western blot identification of prokaryotic expression plasmids pColdⅠ-NP1. (B) Purification of recombinant protein His-NP1. (C) Identification of purified recombinant protein His-NP1 with western blot. (D and E) Western blot identification of eukaryotic expression plasmids pCAGGS-HA-NP1 with anti-NP1 polyclonal and monoclonal antibody anti-HA, respectively. (F) Detection of NP1 protein in PBoV-infected LLC-PK1 cells with anti-NP1.
(TIF)

S1 Table Oligo sequences used for qPCR and PCR. (DOCX)

S2 Table NGS data of microbial species and their relative abundances. (DOCX)

S3 Table The numerical values behind all statistical analyses, graphs, and map. (XLSX)
