# Supplementary figures and images for: Successful in vitro propagation of porcine bocavirus: Demonstrating dual respiratory-enteric tropism and pathogenicity
Source: PLoS Pathog. 2025 Nov 3;21(11):e1013631. doi: 10.1371/journal.ppat.1013631 (PMC12594395; doi:10.1371/journal.ppat.1013631)

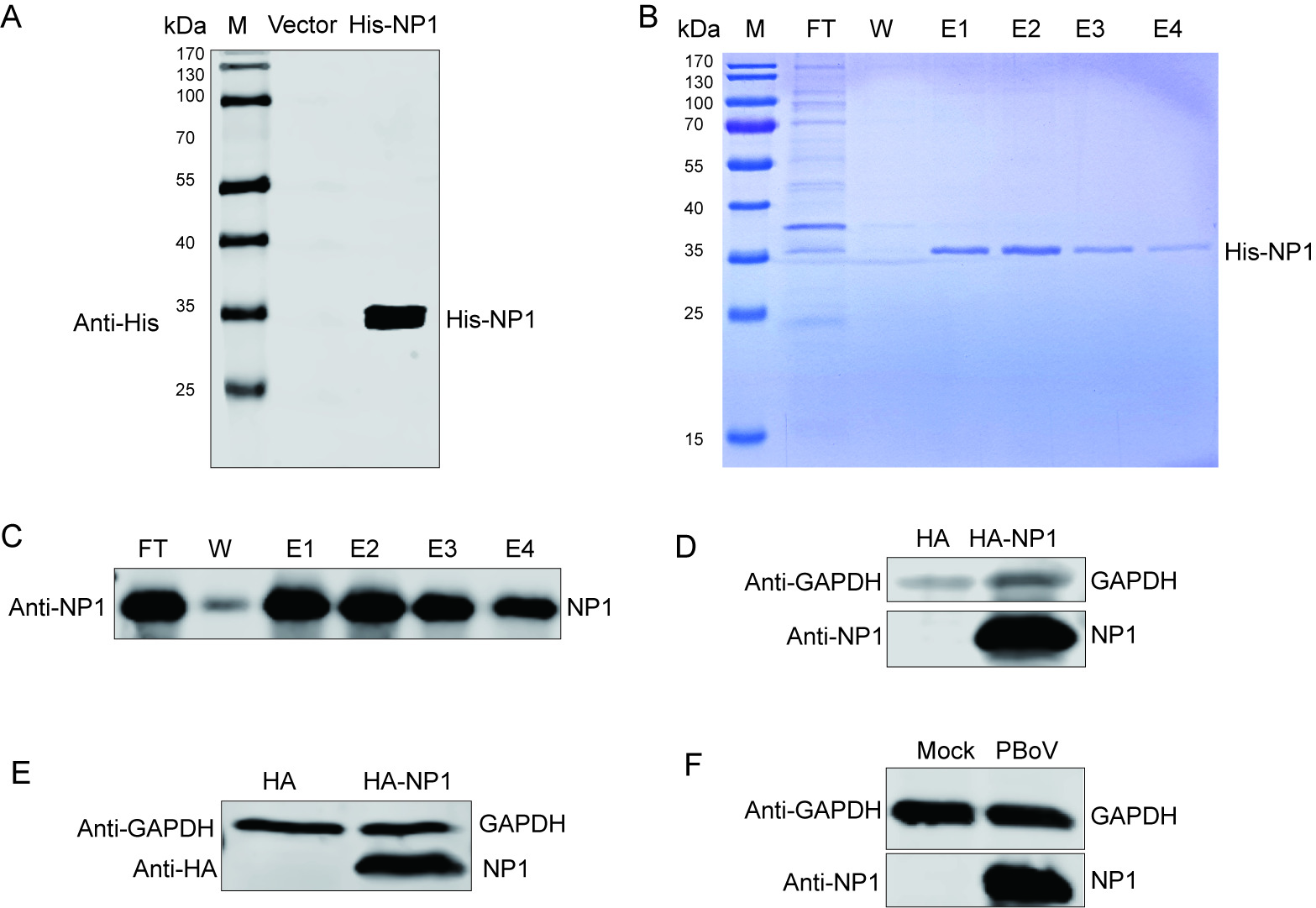

Supplement: S1 Fig — (A) Western blot identification of prokaryotic expression plasmids pColdⅠ-NP1. (B) Purification of recombinant protein His-NP1. (C) Identification of purified recombinant protein His-NP1 with western blot. (D and E) Western blot identification of eukaryotic expression plasmids pCAGGS-HA-NP1 with anti-NP1 polyclonal and monoclonal antibody anti-HA, respectively. (F) Detection of NP1 protein in PBoV-infected LLC-PK1 cells with anti-NP1. (TIF) [file ppat.1013631.s001.tif]
