# Supplementary material for: Successful in vitro propagation of porcine bocavirus: Demonstrating dual respiratory-enteric tropism and pathogenicity
Source: PLoS Pathog. 2025 Nov 3;21(11):e1013631. doi: 10.1371/journal.ppat.1013631 (PMC12594395; doi:10.1371/journal.ppat.1013631)
Supplement: S1 Table — (DOCX) [file ppat.1013631.s002.docx]

**Table S1. Primers used in this study, including primer name and sequence.**

| Primer name | Primer sequence (5’ to 3’) |
| --- | --- |
| PEDV Forward | GTTGAAGAATGGTAAGTTGC |
| PEDV Reverse | ACAGATTGCAAGGTGAAAGG |
| TGEV Forward | AAGGAAGGGTAAGTTGCTCA |
| TGEV Reverse | GGTCCATCAGTTACGCCGAA |
| PoRV Forward | GGCTTTAAAAGAGAGAATTTC |
| PoRV Reverse | GGTCACATCATACAGTTCTAAC |
| PDCoV Forward | TACTCATCCTCAGTTTCGTGG |
| PDCoV Reverse | CTGGTATTTGCCTAACG |
| PBoV Detect Forward | GCTAAGAGGGCTAATCCG |
| PBoV Detect Reverse | CCGCCAAGTGCTGTCTAT |
| PSV Forward | TACAACATAGATGGTGGAG |
| PSV Reverse | CTGGTATTTGCCTAACG |
| PBoV Forward 1 | ATCCAATCATCACTACCCACA |
| PBoV Reverse 1 | CTCTTCGAGCGTTCGAGAC |
| PBoV Forward 2 | CTGCCTGAGGTGGGTGAGA |
| PBoV Reverse 2 | CCGCTGCGAGATGTTGTAT |
| PBoV Forward 3 | AGTGAGTACATCGACTGGAGCAG |
| PBoV Reverse 3 | CCGCCGTGAGATCGTTAGT |
| PBoV Forward 4 | ACCGAGCCGCACGCTTCAGA |
| PBoV Reverse 4 | TCACCCATCACCACCCACA |
| PBoV qPCR Forward | ACAGGCGATAATCAGGAAG |
| PBoV qPCR Reverse | CCAGCCACAGTCAAAGGTA |
| pGAPDH qPCR Forward | ACCTCCACTACATGGTCTACA |
| pGAPDH qPCR Reverse | ATGACAAGCTTCCCGTTCTC |
| hGAPDH qPCR Forward | TCATGACCACAGTCCATGCC |
| hGAPDH qPCR Reverse | GGATGACCTTGCCCACAGCC |
