# Supplementary material for: Successful in vitro propagation of porcine bocavirus: Demonstrating dual respiratory-enteric tropism and pathogenicity
Source: PLoS Pathog. 2025 Nov 3;21(11):e1013631. doi: 10.1371/journal.ppat.1013631 (PMC12594395; doi:10.1371/journal.ppat.1013631)
Supplement: S2 Table — (DOCX) [file ppat.1013631.s003.docx]

| Type | Taxid | Scientific Name | Real Read | Estimate Read | Abundance |
| --- | --- | --- | --- | --- | --- |
| Viruses | 1084715 | Porcine bocavirus 3 | 272445 | 348481 | **65.72%** |
| Viruses | 1131622 | Porcine bocavirus 5/JS677 | 53615 | 99976 | **18.85%** |
| Viruses | 1084717 | Porcine bocavirus 4-1 | 57129 | 77967 | **14.70%** |
| Bacteria | 1264675 | Ralstonia pickettii OR214 | 1811 | 0.34% |  |
| Fungi | 1357677 | Mucor racemosus B9645 | 3 | 1372 | 0.26% |
| Metazoa_Parasite | 670386 | Heterostelium album PN500 | 6 | 654 | 0.12% |
| Viruses | 10345 | Suid alphaherpesvirus 1 | 13 | 13 | 0.00% |

**Table S2. NGS identified microbial species and their relative abundances.**

Shown in bold indicate the relative abundance of bocavirus identified within the virus type.
